# Regulation of Mitotic Cytoskeleton Dynamics and Cytokinesis by Integrin-Linked Kinase in Retinoblastoma Cells

**DOI:** 10.1371/journal.pone.0098838

**Published:** 2014-06-09

**Authors:** William K. A. Sikkema, Arend Strikwerda, Manju Sharma, Kiran Assi, Baljinder Salh, Michael E. Cox, Julia Mills

**Affiliations:** 1 Department of Biology, Trinity Western University, Langley, British Columbia, Canada; 2 Vancouver Prostate Centre, Vancouver Coastal Health Research Institute, Vancouver, British Columbia, Canada; 3 Division of Gastroenterology, University of British Columbia, Vancouver, British Columbia, Canada; 4 Adjunct, Department of Molecular Biology and Biochemistry, Simon Fraser University, Burnaby, British Columbia, Canada; 5 External Associate Member, Department of Cellular and Physiological Sciences, University of British Columbia, Vancouver, British Columbia, Canada; Institut de Génétique et Développement de Rennes, France

## Abstract

During cell division integrin-linked kinase (ILK) has been shown to regulate microtubule dynamics and centrosome clustering, processes involved in cell cycle progression, and malignant transformation. In this study, we examine the effects of downregulating ILK on mitotic function in human retinoblastoma cell lines. These retinal cancer cells, caused by the loss of function of two gene alleles (*Rb1*) that encode the retinoblastoma tumour suppressor, have elevated expression of ILK. Here we show that inhibition of ILK activity results in a concentration-dependent increase in nuclear area and multinucleated cells. Moreover, inhibition of ILK activity and expression increased the accumulation of multinucleated cells over time. In these cells, aberrant cytokinesis and karyokinesis correlate with altered mitotic spindle organization, decreased levels of cortical F-actin and centrosome de-clustering. Centrosome de-clustering, induced by ILK siRNA, was rescued in FLAG-ILK expressing Y79 cells as compared to those expressing FLAG-tag alone. Inhibition of ILK increased the proportion of cells exhibiting mitotic spindles and caused a significant G2/M arrest as early as 24 hours after exposure to QLT-0267. Live cell analysis indicate ILK downregulation causes an increase in multipolar anaphases and failed cytokinesis (bipolar and multipolar) of viable cells. These studies extend those indicating a critical function for ILK in mitotic cytoskeletal organization and describe a novel role for ILK in cytokinesis of *Rb* deficient cells.

## Introduction

Integrin linked kinase (ILK) is a protein well established for its role in proliferation [1]–[4] and cancer biology [5]–[7]. ILK is upregulated in human retinoblastomas (Rb)[3], a retinal tumour caused by the loss of function of two gene alleles (*Rb1*) that encode the first cloned tumour suppressor [8]. Because this form of eye cancer arises from mutations to both copies of the *Rb* gene, this tumour suppressor was given the same name as the cancer that it caused when it was mutated. It has been subsequently found that loss of the Rb tumour suppressor function is a common phenomenon in many types of cancer and that patients that inherit mutations in the Rb tumour suppressor gene are at a much higher risk of developing other cancers throughout their lifetime [9]. Although the transformation of retinal cells and the development of tumours are not fully understood, the progression of this cancer is considered intimately related to deficient Rb signaling, increased and inappropriate proliferation and the ability to survive mitotic infidelity [10], [11]. ILK drives the proliferation of human retinoblastoma cells [3]and is a key regulator of G1/S cyclin-cdk activities [4], [12], a critical step in the Rb signaling pathway. Furthermore, in cells containing a functional *Rb* gene, ILK directly regulates its activity [4], [12]. To date, ILK's role in cells in which the Rb tumour suppressor gene is inactivated, has not been studied in detail.

During cell division ILK has been shown to regulate microtubule dynamics and centrosome clustering, processes involved in cell cycle progression and malignant transformation [13]–[18]. ILK is required for centrosome clustering in several breast and prostate cancer cells with supernumary centrosomes [16]. These cancer cell lines are more sensitive to ILK inhibition than cells with two centrosomes [16]. ILK localizes to the centrosomes and regulates microtubule organization during mitosis. ILK-interacting proteins at the centrosome regulate centrosome clustering. Specifically, ILK influences Aurora A/ch-TOG/TACC3 complex formation, protein interactions essential for mitotic spindle assembly and mitosis [14], [16].

ILK depletion has resulted in mitotic defects in a number of cells including Drosophila S2 cells, mouse hepatocytes and human brain, breast, prostate and cervical cancer cells [15], [16], [19]–[22]. ILK loss resulted in mitotic arrest [15], [21], [22] and either subsequent exit from mitosis or cell death [16]. An increase in multinucleated cells was not reported. In contrast, we show that ILK inhibition in retinoblastoma cells, markedly increases the percentage of multinucleated cells, an effect that correlates with altered mitotic spindle organization and failed cytokinesis.

## Results

### Downregulation of ILK Increases Nuclear Size and Multinucleated Cells

**Figure 1:**
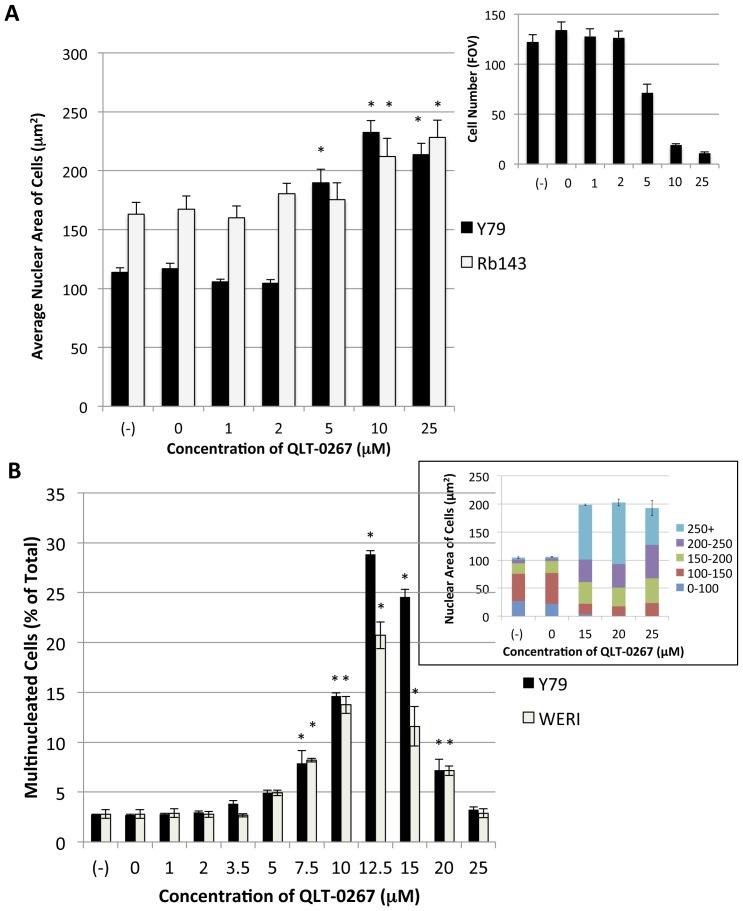
QLT-0267 Increases Nuclear Area and Multinucleated Cells in a Concentration-Dependent Manner. (A) Changes in nuclear size and nuclear number in retinoblastoma cell lines. Y79 and Rb143 cell cultures were exposed to increasing concentrations of QLT-0267, and a dose-dependent increase in size was observed (n = 3–4). Controls lacking drug vehicle, labeled (-), or with drug vehicle alone, labeled 0, were also included. Size is measured as a unit of Hoechst-stained area by Metamorph Premier software. An ANOVA followed by Dunnett's *post hoc* test was used to determine the significance of observed differences. Data are represented as the mean ± SEM and represent three independent experiments; total cells sampled were greater than 1400 cells/cell line (*p<0.05, different from nonvehicle control). (Inset) A corresponding decrease in total Y79 cell number was also observed with the increase in nuclear cell area. The bar graph depicts the average Y79 cell number per field of view (FOV) with increasing QLT-0267 concentration. (B) Since size varied more dramatically in Y79 cells, these cells were analyzed for changes in nuclear number (n = 3). Weri-Rb 27 cells were also analyzed. The changes in multinucleated cells tracked with changes in size for Y79 cells. A maximum concentration effect was observed at 12.5 µM for both cell lines and a surprising decrease in multinucleated cells was observed at 20 and 25 µM (relative to 12.5 µM). Controls lacking drug vehicle, labeled (-), or with drug vehicle alone, labeled 0, were also included. 10 µM was used as the working concentration for further analysis. An ANOVA followed by Dunnett's *post hoc* test was used to determine the significance of observed differences. Data are represented as the mean ± SEM from three independent experiments with greater that 200 cells/treatment (*p<0.05, different from nonvehicle control). (Coloured Inset) To further analyze the apparent disparity between the effects of high concentrations of QLT-0267 on the average nuclear area and the percentage of multinucleated cells, flattened stacks of Y79 cells were analyzed (total sample size was 45 000 cells from 3 independent experiments). The proportion of cells having a nuclear area up to and including 100 (0–100), <100≤150 (100–150), <150≤200 (150–200), <200≤250 (200–250) and >250 (250+) was determined for the controls and cells treated with 15–25 µM QLT-0267.

To determine the concentration of ILK inhibitor that compromised cytokinesis in retinoblastoma cells, a concentration-dependent effect on the nuclear area of retinoblastoma cells was obtained for QLT-0267 or vehicle control (DMSO) over a 5 day period. QLT-0267 is a selective, small molecule inhibitor belonging to the K15792 class of the pharmacor family [22]–[24]. Cells were also treated in the absence of DMSO or drug (labeled (-)), or with DMSO alone (labeled 0), as a control for drug vehicle. The IC50 for ILK kinase activity is between 2 and 5 µM QLT-0267 depending on the cell type [23]. This correlates well with the effect on nuclear size (Fig. 1A) supporting a role for ILK kinase activity in the multinucleated phenotype. Nuclear area was seen to increase most dramatically in Y79 cells as compared to Rb143 cells. In Y79 cells, the average nuclear area following a 5 day exposure to 10 µM was 110 µm^2^ above vehicle control versus 45 µm^2^ above vehicle control in Rb143 cells (Fig. 1A). A corresponding decrease in total Y79 cell number was also observed with the increase in nuclear cell area. This is evident by the inset (Fig. 1A) depicting the average Y79 cell number per field of view (FOV) with increasing QLT-0267 concentration. Because the increase in nuclear size was most dramatic for Y79 cells, a concentration-dependent effect for nuclear number was determined in the Y79 cell line. In controls lacking drug vehicle (labeled (-)) or DMSO vehicle controls (labeled 0), we observed a low incidence of multinucleated (≥2) cells over 5 days. In contrast, cells exposed to 10 and 12.5 µM QLT-0267 exhibited 15% and 29% multinucleated cells, respectively (Fig. 1B). A concentration-dependent effect for nuclear number was also determined for Weri-Rb27 cells. As for Y79 cells, a low incidence of multinucleated (≥2) cells over 5 days was observed in control groups while cells exposed to 10 and 12.5 µM QLT-0267 exhibited the highest percentage of multinucleated cells (being 14% and 21%, respectively). While a maximum effect on multinucleation was observed at 12.5 µM for both cell lines, a surprising decrease in multinucleated cells was observed at higher concentrations (15–25 µM) relative to 12.5 µM. To further address the apparent disparity between the effects of high concentrations of QLT-0267 on the average nuclear area and the percentage of multinucleated cells, flattened stacks of Y79 cells were analyzed for the proportion of cells having a nuclear area, in µm, up to and including 100 (0–100), <100≤150 (100–150), <150≤200 (150–200), <200≤250 (200–250) and >250 (250+). Relative to control lacking drug vehicle (-) and DMSO control (0) there was an increase in the proportion of cells having an average nuclear area greater than 150 µm when QLT-0267 was exposed to cells at higher concentrations (15–25 µM). Conversely, a decrease in cells less than or equal to 150 µm was also observed. This data indicates that the ILK inhibitor, at the highest concentration, increases the proportion cells having large nuclei without increasing the percentage of multinucleated cells. Differences in the concentration-effect of QLT-0267 in Y79 cells between the average nuclear area and multinucleation may suggest that replication of the nuclear genome was occurring in the absence of karyokinesis at higher concentrations of QLT-0267.

**Figure 2:**
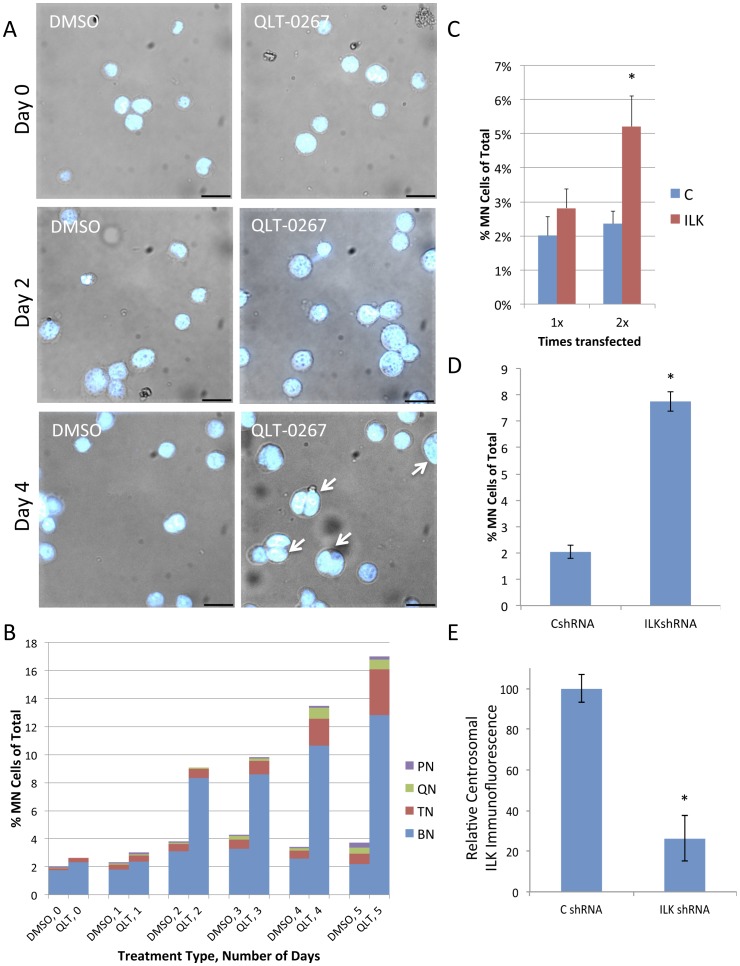
Loss of ILK and ILK Inhibition Increases Multinucleated Cells Over Time. (A) Cells were fixed and stained with Hoechst to visualize binucleated (BN), trinucleated (TN), quadrinucleated (QN) and poly-nucleated (PN; >4 nuclei) over days cultured. An increase in cells with ≥2 nuclei or multinucleated (MN) can be seen in Y79 cells exposed to 10 µM QLT-0267 for 0, 2, 4 days in culture. White arrows depict cells that are multinucleated as determined by scanning through individual z-stacks. Scale bar, 40 µm. (B) In the presence of QLT-0267, mitotic cells give rise to multinucleated cells over time. Cells were fixed and stained with Hoechst to visualize bi-nucleated, tri-nucleated, quadri-nucleated and poly-nucleated cells (>4 nuclei) over days cultured. The percentage of control cells (DMSO) or QLT-0267 (10 µM) with more than one nucleus was determined after fixing and staining at 0-5 days after treatment. The percentage of multinucleated cells (≥2 nuclei) was calculated for a minimum of 300 cells. The data represent 3 separate experiments. (C) Y79 cells were treated with control (C) or ILK siRNA 1x or 2x. Histograms indicate the percentage of multinucleated cells in control and siRNA-treated cells. Data are mean ± SEM from three (1x transfected) or four (2x transfected) independent experiments in which >450 total cells/trial were analyzed. * p<0.05, different from C by a Student t-test. (D) Y79 cells were transduced with either control shRNA or ILK shRNA lentivirus. Histograms indicate the percentage of multinucleated cells. Data are mean ± SEM from three independent experiments. * p<0.01, different from C by a Student t-test.

As observed effects may be due to perturbed mitoses that led to the accumulation of a population of multinucleated cells, subsequent analysis with the ILK inhibitor will include both early (i.e. 24 hours) and late (i.e. 5 days) time points. To determine the effect of QLT-0267 on the accumulation of nuclear number over time, retinoblastoma cells were examined over 5 days (Fig. 2A,B). In control cells (DMSO, 0; QLT, 0), we observed a relatively constant and low incidence of binucleate cells (1.7–3.3%) and cells with ≥3 nuclei (0.3–1.5%) (Fig. 2B). In contrast, cells exposed to QLT-0267 exhibited a steady increase in the number of cells with 2 or more nuclei or multinucleated (labeled MN), over the course of the experiment. Early on, the most dramatic change was seen in binucleate cells (labeled BN) of QLT-0267 treated cells, increasing from 2.4% (day 1) to 8.3% (day 2) and then to 13.0% (day 5). This was followed by a delayed but equally dramatic increase in cells having ≥3 nuclei, increasing from 0.8% (day 2) to 4.2% (day 5). The accumulation of binucleate cells first, followed by cells with ≥3 nuclei (including trinucleated, quadrinucleated and polynucleated cells), suggest that cells having 3 or more nuclei are derived from binucleate cells.

Although QLT-0267 is reported to be a highly selective ILK inhibitor [23], it is still possible that changes in nuclei number were a result of off-target effects. Alternatively, evidence exists that ILK is a pseudokinase [25], making it important to study the functional downregulation of ILK by other means. Therefore, we used ILK siRNA to decrease ILK expression as previously described [15]. Briefly, to achieve sufficient ILK knockdown, cells were transfected twice (at day 0 and 2). The percentage of multinucleated cells (≥2 nuclei) two days following transfection (1x and 2x) indicates that there was a significant increase in the percentage of multinucleated cells after two transfections. Multinucleation increased to 5.2%±0.9% SEM as compared to 2.4%±0.4% SEM in cells treated with control siRNA (Fig. 2C). Temporally speaking, this effect can be compared to the approximate doubling of multinucleation above control seen for 10 µM QLT-0267 at two days of treatment. As an alternate method for downregulating ILK expression, Y79 cells were transduced with control shRNA or ILK shRNA Lentiviral Particles. Virally transduced cells were subsequently selected in puromycin containing media for >3 weeks. Analysis of nuclei number indicates that multinucleated cells in ILK shRNA transduced cells were 7.7%±0.4% SEM as compared to 2.1%±0.3% in control cells, n = 3 (Fig. 2D). To ensure that ILK expression was knocked down, we measured the fluorescence intensity profile of ILK using Metamorph imaging software and compared staining of ILK at the centrosomes. ILK intensity was decreased to 26.1%±10.5% of control, n = 3 in these same cultures (Fig. 2E).

### ILK Inhibition Results in Mitotic Spindle Disorganization and Altered F-Actin Architecture

**Figure 3:**
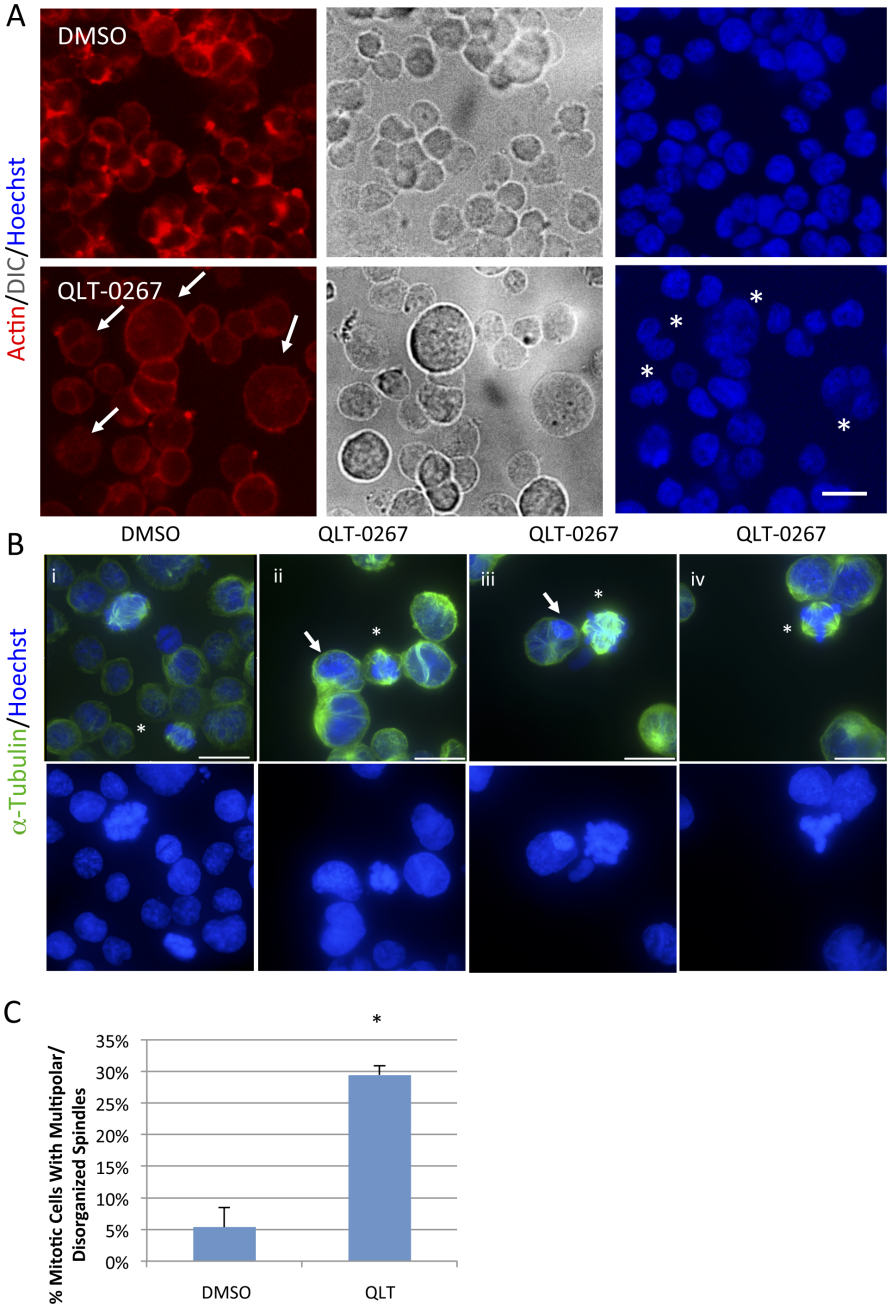
Treatment with the ILK Inhibitor QLT-0267 Results in Aberrant Cytokinesis and Mitotic Spindle Disorganization. (A) A dramatic decrease in F-actin staining was observed in Y79 cells after a 5-day incubation to 10 µM QLT-0267 (n = 4). Note the increase in overall cell size following ILK inhibition as evident by DIC and F-actin staining (see large cells labeled by arrows). Also, cells that are labeled with asterisks are multinucleate in QLT-0267 treated Y79 cells (Fig. 3A). Both an increase in cell size and multinucleation are consistent with a cytokinesis defect occurring in ILK inhibited cells. Scale bar, 20 µm. (B) Y79 cells were treated with 10 µM QLT-0267 for 5 days and stained as indicated (upper panel show composites of α-tubulin and Hoechst stained cells; Lower panel show only Hoechst staining of these same cells). A tetrahedral configuration of spindle was most commonly seen in multipolar cells within the QLT-0267 treated group (see cells labeled by asterisks). Multipolar spindles were observed in mitotic QLT-0267 treated cultures as early as 7 hours after exposure (data not shown). DNA of cells just beginning to condense are labeled by arrows. The cell in ii is condensing in all nuclei while the one in iii is condensing in only one nuclei indicating asynchronous nuclear division in QLT-0267 treated cultures. Scale bar, 20 µm. (C) Multipolar/Disorganized spindles were assessed in mitotic retinoblastoma cells following a 3–5 day treatment with QLT-0267 or DMSO control. Cells were fixed and stained with Hoechst and α-tubulin to assess mitotic cells with aberrant spindles. The bar graph represents the percentage of mitotic cells that display multipolar/disorganized spindles and is expressed as mean ± SEM. A significant increase in aberrant spindles was observed in Y79 cells treated with the ILK inhibitor as compared to DMSO control. * p<0.005 as determined by Student's t-test, n = 12; total sample size was greater than 300 cells.

Cortical F-actin staining is altered in cells lacking ILK or in which ILK has been inhibited [18], [24]. ILK inhibition resulted in a marked reduction in cortical F-actin (Fig. 3A). DIC analysis of F-actin stained cells indicate that there are a much higher number of unusually large Y79 cells in QLT-0267 treated groups (see cells labeled with an arrow). Also, cells that are labeled with asterisks are clearly multinucleate in QLT-0267 treated Y79 cells (Fig. 3A). Both observations are consistent with a cytokinesis defect occurring in ILK inhibited cells.

Inhibition of ILK activity has been shown to disrupt mitotic spindle function (including spindle organization) in HEK 293 cells, HeLa cells, breast and prostate cancer cells [15], [16]. To address the role of ILK kinase activity in the mitotic spindle organization of retinoblastoma cells, Y79 cells were exposed to 10 µM QLT-0267 and then fixed and immunostained for α-tubulin and Hoechst. A tetrahedral configuration of spindle was most commonly seen for cells undergoing a multipolar division in the QLT-0267 treated group (Fig. 3B). Multipolar spindles were observed in mitotic QLT-0267 treated cultures as early as 7 hours after exposure (data not shown). Multipolar mitotic spindles were only rarely observed in vehicle-treated retinoblastoma cells. Quantitation of mitotic cells with multiple/disorganized spindles indicate that there was a significant increase in aberrant spindles observed in Y79 cells treated with the inhibitor. QLT-0267 exposure resulted in 29.4% of cells displaying multipolar spindles versus 5.4% in vehicle treated cells (Fig. 3C). These data are consistent with earlier reports showing that ILK activity is required for the maintenance of spindle integrity during mitosis in a wide variety of cancer cell lines [15], [16].

**Figure 4:**
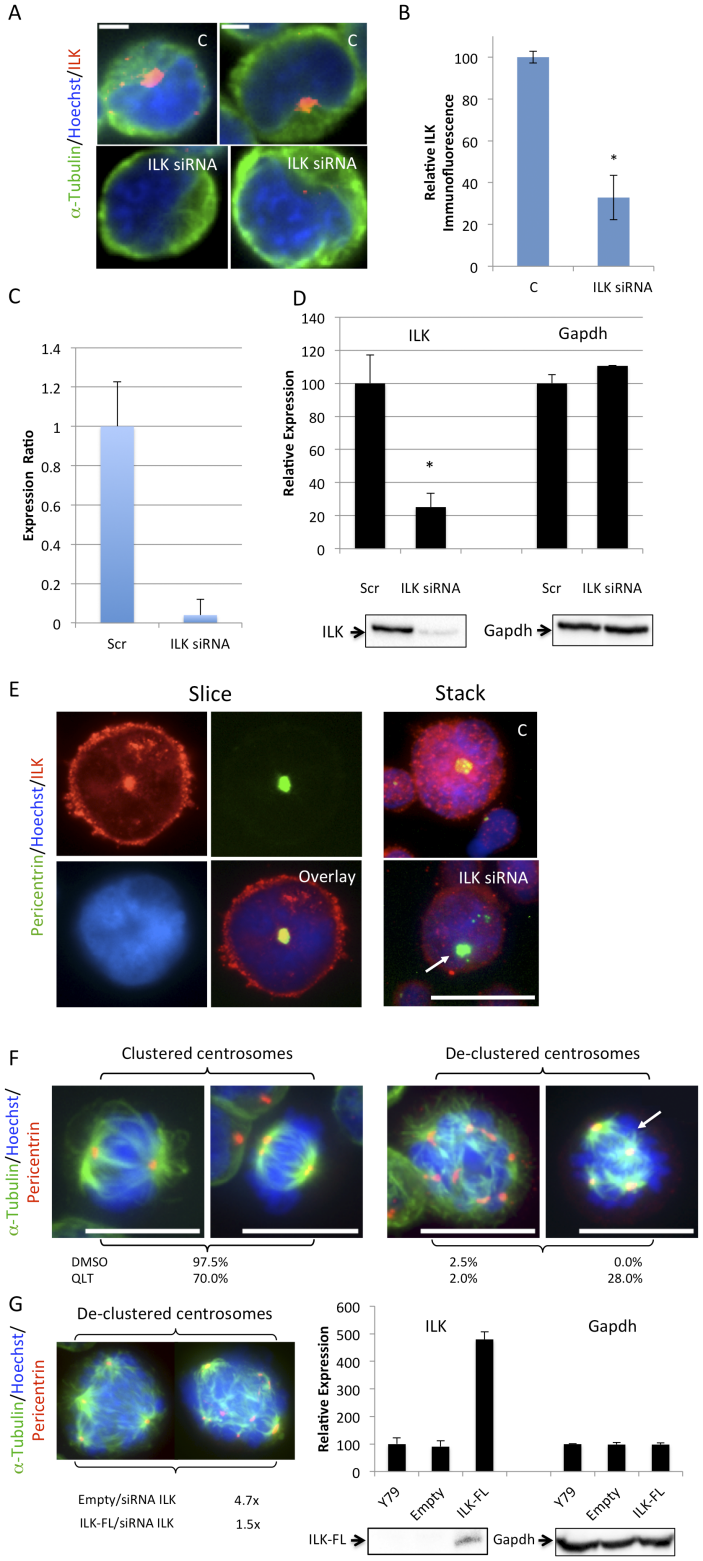
Loss of ILK Causes an Increase in Mitotic Cells Displaying Multipolar Division and De-clustered Centrosomes. (A) ILK immunofluorescence co-localized with α-tubulin at the centrosomes. Supernumerary centrosomes are typically clustered in one location of the cell when retinoblastoma cells are interphase. Y79 cells were treated with control (C) or ILK siRNA. A marked reduction of ILK centrosomal staining was observed when cells were exposed to ILK siRNA as compared to control siRNA. Shown are representative figures used in the analysis of relative centrosomal ILK immunofluorescence (see Figure 4B) where a predetermined threshold permitted only the brightest of pixels to be visible in a given field of view. Scale bar, 4 µm. (B) Staining of ILK at the centrosomes was estimated by comparing the fluorescence intensity profile of ILK at the centrosomes using Metamorph imaging software. For this analysis, a threshold was determined, allowing only the brightest of pixels to be visible in a given field of view in the control siRNA. Histograms indicate the relative intensity of ILK immunofluorescence at the centrosomes following treatment with control (C) or ILK siRNA. Data represent 3 separate trials calculated for a minimum of 500 cells/trial, p<0.05. (C) Integrin-linked kinase (ILK) knockdown using small interfering RNA (siRNA) in Y79 cells. Y79 cells were transfected with either a targeted oligo for ILK or a non-specific, scrambled control. After two, consecutive transfections, the total RNA was extracted and the cDNA was used for quantitative PCR analysis. Samples were run in triplicate from three separate trials, and the results were corrected for the β-actin data run each time. Bar graph depicts ILK RNA expression levels of ILK siRNA treated cells (ILK siRNA) relative to scrambled control siRNA (Scr) in a representative trial, run in triplicate. Data represent the average ± SD. (D) Western blots of Y79 cell lysates following treatment with ILK siRNA or scrambled control (Scr) was performed. Blots were probed first with a mouse anti-ILK antibody then stripped and reprobed with a rabbit anti-Gapdh antibody. Bar graph represents densitometric analysis of blots probed for ILK and Gapdh. Protein expression for ILK and Gapdh from 3 independent trials were averaged and normalized to the scrambled control. Data are mean ± SEM. * p<0.05 as determined by Student's t-test. Representative Western blots of ILK and Gapdh are also shown below each bar graph. (E) (Left “Slice”) Y79 cells were costained for ILK together with pericentrin and Hoechst. An overlay of these individual stains shows clear colocalization of ILK with pericentrin at the centrosomes. (Right “Stack”) Y79 cells treated with C or ILK siRNA that were subsequently costained for ILK and pericentrin. Flattened stacks showed a marked reduction of ILK centrosomal staining. Control (C) and ILK siRNA treated sister cultures are shown and are representative of three independent experiments. ILK staining has been reduced at the centrosome labeled with a white arrow in ILK siRNA treated cells. (F) Y79 cells were treated with 10 µM QLT-0267 for 5 days and then fixed and stained for α-tubulin and pericentrin. Cells with multipolar spindles in the DMSO treated group displayed highly disorganized α-tubulin staining and had large numbers of spindles. Conversely, in the presence of the ILK inhibitor, prominent α-tubulin staining appeared only associated with spindles and multipolar spindles were arranged most often in a tetrahedral configuration. The spindle pole labeled with a white arrow projects away from the plane of the paper while the others run more parallel with it. Bar represents 30 µm. (G) Centrosome de-clustering was also measured in Y79 cells expressing FLAG-tag (labeled empty) or FLAG-ILK constructs (labeled ILK-FL) following knockdown of ILK with siRNA. This rescue experiment was performed to validate the ILK siRNA centrosome de-clustering effect. After transfection, FLAG expressing clones were selected for 10 days and then treated with ILK siRNA or scrambled control. De-clustered centrosomes were compared in mitotic cells following ILK siRNA treatment and results were normalized to scrambled control in each experimental group. De-clustering of centrosomes in mitotic cells expressing FLAG-tag and treated with ILK siRNA was 4.7x that seen in scrambled control while in cells expressing FLAG-ILK de-clustering was 1.5x that seen in scrambled control. Data represent three independent experiments in which ≥100 mitotic cells were sampled for each condition. Y79 cell extracts untransfected, or transfected with either FLAG-tag or FLAG-ILK were analyzed by Western blotting. Blots were probed first with a mouse anti-FLAG antibody and subsequently reprobed with a rabbit anti-Gapdh antibody. Western blots of FLAG-ILK (labeled ILK-FL) and Gapdh are shown. Bar graph represents samples, run in quadruplicate. Data are expressed as the average ± SEM.

ILK localizes to the centrosome and plays a critical role in centrosome clustering, an action that presumably circumvents multipolar mitosis and cell death. Indeed cancer cell lines with a high percentage of supernumerary centrosome-harboring cells were more vulnerable to survival effects associated with de-clustered centrosomes [16]. Supernumerary centrosome-harbouring cells were observed in Y79 cell lines (Fig. 4). Co-staining cells with a polyclonal anti-ILK antibody and an anti-α-tubulin antibody that stains the mitotic spindle indicate that ILK localizes to the centrosomes in retinoblastoma cells (Fig. 4A). Shown are representative figures used in the analysis of relative centrosomal ILK immunofluorescence (see Fig. 4B) where a predetermined threshold permitted only the brightest of pixels to be visible in a given field of view. Following ILK knockdown with siRNA, ILK staining is significantly decreased at the centrosomes (Fig. 4A,B) relative to a non-specific, scrambled control. Expression levels of ILK siRNA treated cells relative to scrambled control siRNA were determined using quantitative PCR analysis. Total ILK RNA expression levels of samples were run in triplicate from three separate trials. A bar graph depicts ILK RNA expression levels of ILK siRNA treated cells (ILK siRNA) relative to scrambled control siRNA (Scr) (Fig. 4C). We also determined protein expression levels of ILK in ILK siRNA treated cells relative to scrambled control siRNA. Densitometric analysis of Western blots probed for ILK indicate that ILK siRNA treated cells expressed significantly less ILK protein (25.1%±8.4) relative to scrambled control siRNA (100%±17.2). Gapdh expression in these same trials was not different from control indicating that the observed differences were not due to a loading error or nonspecific effects of siRNA treatment (Fig. 4D). To ensure that ILK staining is indeed centrosomal in retinoblastoma cells, Y79 cells were costained for ILK together with pericentrin and Hoechst. An overlay of these individual stains shows clear colocalization of ILK with pericentrin at the centrosomes (see Fig. 4E “Slice”). To determine whether or not centrosomal ILK decreased following ILK siRNA treatment, Y79 cells treated with control or ILK siRNA were subsequently costained for ILK and pericentrin. ILK immunofluorescent staining without a predetermined threshold is included (see Fig. 4E “Stack”). A marked reduction of ILK centrosomal staining was observed following ILK downregulation. A control (C) and their corresponding ILK siRNA treated sister cultures are shown (Figure 4E). ILK staining was reduced at the centrosome labeled with a white arrow in ILK siRNA treated cells. It is also noteworthy that in ILK downregulated cells, ILK expression is not just decreased at the centrosome (Figure 4E). Rather, in immunofluorescent images without a predetermined threshold, a marked reduction in ILK immunofluorescence is observed throughout the cell (Figure 4E). Y79 cells were treated with QLT-0267 for 5 days and then fixed and stained for α-tubulin and pericentrin to quantitate the cells displaying de-clustered centrosomes when cells were in either prometaphase or metaphase (Fig. 4F). De-clustered centrosomes were further divided into those having a tetrahedral arrangement and those without. Treatment with the ILK inhibitor resulted in 30% of the cells displaying de-clustered centrosomes (as compared to 2.5% in the DMSO control group). Most of those de-clustered cells were in the tetrahedral arrangement. Although de-clustered cells were also observed in control cells these presented in a nontetrahedral arrangement. Centrosome de-clustering was also measured following knockdown of ILK with siRNA. This was performed in Y79 cells expressing FLAG-tag (labeled Empty) or FLAG-ILK (labeled ILK-FL) in order to validate the ILK siRNA centrosome de-clustering effect as being specific to ILK downregulation (and not an off-target effect). De-clustering of centrosomes in mitotic cells expressing FLAG-tag and treated with ILK siRNA was 4.7x that seen in scrambled control while in cells expressing FLAG-ILK de-clustering was 1.5x that of scrambled control (Fig. 4G). Lysates of Y79 cells alone, or transfected with either FLAG-tag or FLAG-ILK were analyzed by Western blotting to confirm that the Y79 cells were indeed expressing FLAG-tagged ILK. Western blots were probed first using an anti-FLAG antibody (to detect FLAG-ILK) and subsequently reprobed with an anti-Gapdh antibody (to ensure that observed differences were not due to a loading error).

### ILK Inhibition Results in Decreased Cell Proliferation, Mitotic Arrest and Differential Effects on Bipolar and Multipolar Cytokinesis

**Figure 5:**
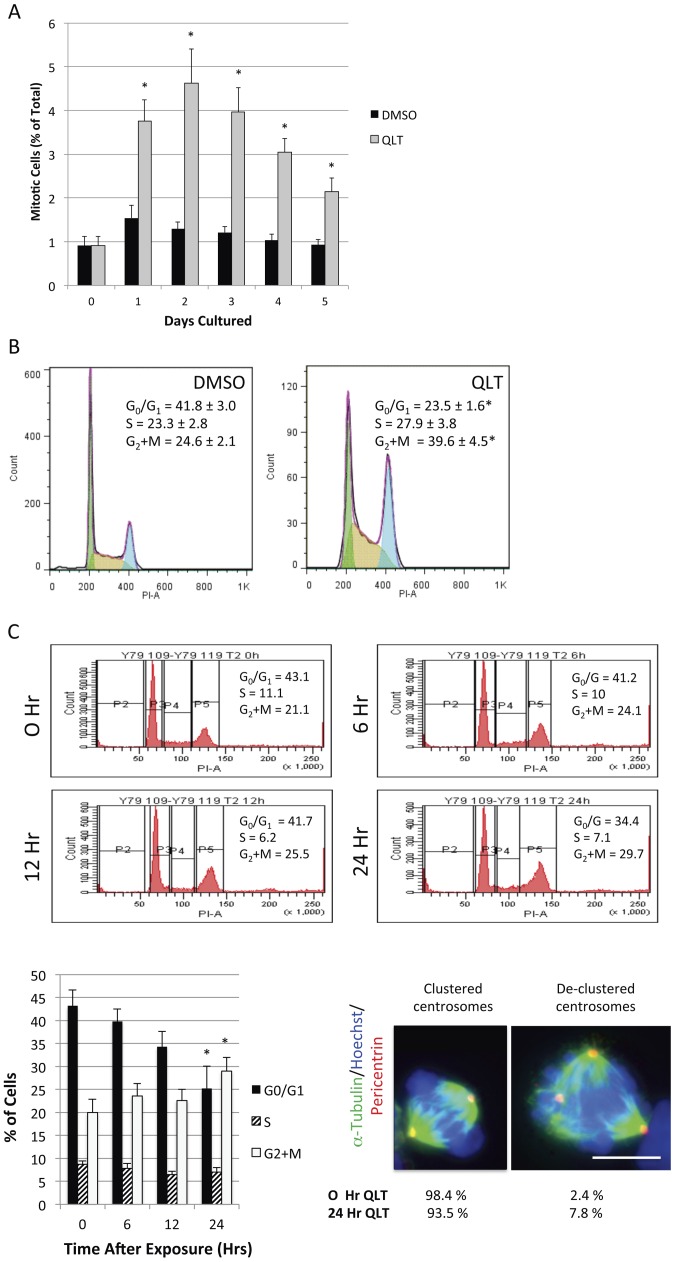
The ILK Inhibitor QLT-0267 Results in Cell Cycle Arrest. (A) Y79 cells were plated in media containing 10 µM QLT-0267 or DMSO and fixed at 0-5 days in culture. The percentage of cells exhibiting mitotic spindles was determined in cells visualize by tubulin immunofluorescence and Hoechst staining. The QLT-0267 treated Y79 cells had a greater percentage of mitotic cells at 1-5 days than the DMSO treated population. Data are mean ± SEM of eight separate experiments in which >300 cells/day/trial were counted for each condition. (B) The ILK inhibitor QLT-0267 induces G_2_/M cell cycle arrest following a long-term exposure. Y79 cells were exposed to 10 µM QLT-0267 or vehicle control for 5 days. Cells were then stained with propidium iodide for the fluorescence-activated cell sorting analysis. A marked increase in the percentage of Y79 cells in the G_2_+M-phase was observed in QLT-0267 treated cells as compared to vehicle control. The percentage of cells in G_0_/G_1_-, S- and G_2_+M-phase is expressed as an average ± SEM of 5 independent platings (n = 5) for both QLT-0267 and vehicle treated groups. * p<0.05 as determined by Student's t-test. (C) ILK inhibition induces G2/M arrest and de-clustered centrosomes 24 hours after treatment. Cell cycle progression was determined by flow cytometric analysis of PI stained Y79 cells at 0, 6, 12 and 24 hours after treatment with drug. The bar graph depicts the percentage of cells ± SEM in G_0_/G_1_-, S- or G_2_+M-phase at each time point. An ANOVA followed by Dunnett's *post hoc* test was used to determine the significance of observed differences between the cell cycle phases at 0 as compared to all other time points (* p<0.05). Data are represented as the mean ± SEM, n = 4-5 independent experiments. A separate population of cells were also fixed and stained for α-tubulin and pericentrin at 0 and 24 hours after drug exposure. An increase in mitotic cells undergoing multipolar division and de-clustered centrosomes was observed at 24 hours (relative to 0 hours). Percentages were determined from 3 to 4 independent trials. Calibration bar represents 8 µm.

In other cancer cell lines (including breast, cervical, prostate or brain cancer cells), ILK loss resulted in mitotic arrest [15], [16], [21], [22]. Time-lapse microscopy using breast cancer cells indicated that following mitotic arrest ILK deficient cells subsequently exited from mitosis or died [16]. These studies have shown that the mitotic index of cancer cells is increased by at least two fold when cells are exposed to 5-10 µM of QLT-0267. This has been shown for cells treated with the inhibitor for only 7 hours. The fraction of retinoblastoma cells with mitotic spindles was determined over 5 culture days in cells visualized by tubulin immunofluorescence and Hoechst staining. We determined mitotic cells by scoring cells having chromosomal condensation and spindle formation microscopically. A greater than twofold increase in mitotic cells was observed over 1-5 days in QLT-0267 treated cultures as compared to vehicle controls (Fig. 5A). To examine the effect of ILK inhibition on cell cycle progression we stained fixed Y79 cells with propidium iodide and performed fluorescence-activated cell cycle analysis. A significant increase in the fraction of Y79 cells in G_2_+M-phase was observed following a 5-day QLT-0267 treatment (Fig. 5B). The percentage of cells in G_2_+M-phase was 39.6±4.5% as compared to 24.6±2.1% in vehicle control (see Fig. 5B Inset). As the percentage of cells in G_2_+M-phase increased, the percentage of cells in G_1_/G_0_-phase decreased, while the proportion of cells in S-phase was not significantly different. We next performed a time trial to determine the earliest time point at which QLT-0267 increased G2/M arrest. Cell cycle progression was determined by flow cytometric analysis at 0, 6, 12 and 24 hours after treatment with the drug. A significant change in the percentage of cells in G_2_+M- and G_1_/G_0_-phase occurred at 24 hours as compared to 0 hours (earlier exposure times were not significantly different from 0 hours). Conversely, the proportion of cells in S-phase did not differ between 0 and all other time points. A separate population of Y79 cells were also fixed and stained for α-tubulin and pericentrin at 24 hours after drug exposure and percentages of mitotic cells undergoing multipolar division were determined from three independent trials. An modest increase in mitotic cells undergoing multipolar division and de-clustered centrosomes as was observed in cells treated with 10 µM QLT-0267 for 24 hours as compared to the increase observed following a 5 day treatment (compare percentages shown in Fig. 4F with those in Fig. 5C (lower right panel)). Specifically, de-clustered centrosomes were observed in 7.8% of mitotic cells following a 24 hour exposure to the ILK inhibitor and in 30.0% of mitotic cells following a 5 day exposure.

**Figure 6:**
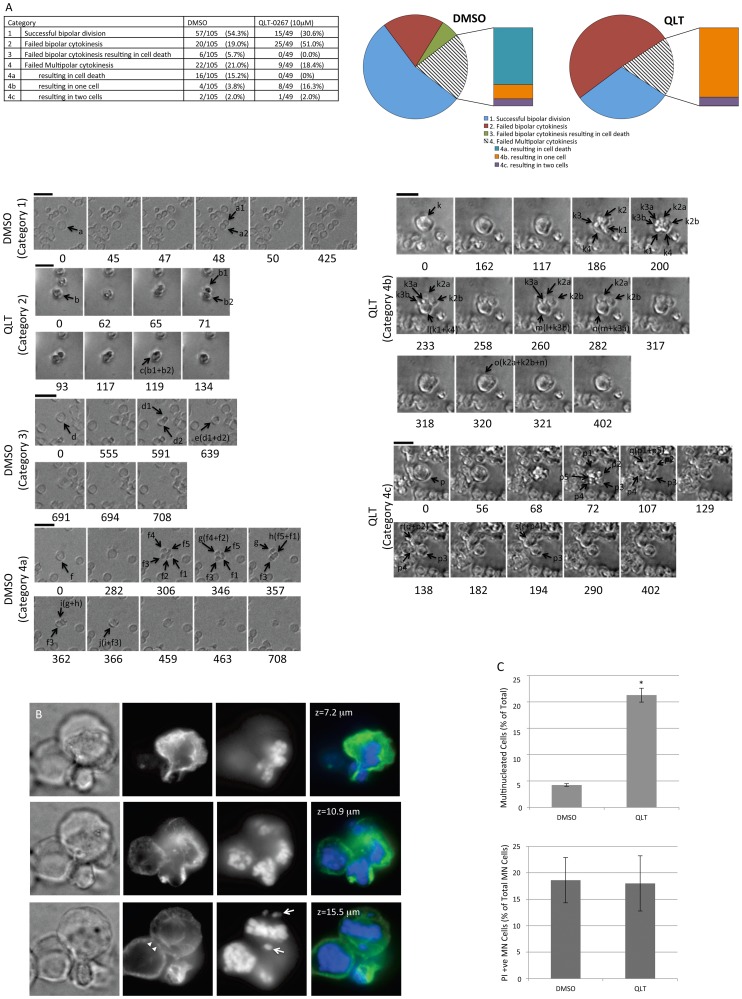
Inhibition of ILK Decreases Bipolar and Multipolar Cytokinesis while Increasing Survival of Multinucleated Cells. (A) Y79 cells were treated with either DMSO or 10 µM QLT-0267 24 hours before imaging began and real-time microscopy was performed for 12 hours with images acquired every 1.5 minutes. Movies were observed to identify cells undergoing mitosis. All mitotic cells were categorized into four main groups as shown in the table and pie chart. Failed multipolar cytokinesis was further divided into 3 subcategories (4a-c). Subcategories represent the number of cells that were produced by failed multipolar divisions and the number of failed multipolar divisions leading to death. Multipolar divisions not leading to cell death were categorized as “failed” if their polarity of division (number of poles in a multipolar mitosis) during anaphase was greater than the number of cells produced. Interestingly, multipolar cytokinesis leading to death did not occur in retinoblastoma cells treated with the ILK inhibitor while 16% of cells undergoing multipolar cell division in DMSO treated cultures died (see Category 4a). However, a much higher percentage of multinucleated cells in QLT-0267 treated cells exited mitosis without completing cytokinesis as compared to controls (Category 4b: 16% versus 4%, respectively). Failed bipolar division was also increased in QLT-0267 treated Y79 cells as compared to DMSO controls (Category 2: 51% versus 19%, respectively). Categories defined in the table and pie chart are also shown as frames from phase movies. Numbers under each frame indicate the length of time in minutes from the initiation of mitosis (as indicated by the cell starting to round up). DMSO (Category 1): Represent a cell treated with DMSO that undergoes successful bipolar division. Within 50 minutes it has completed cytokinesis (frame 5, 50 min) giving rise to two cells (a1 and a2). The final frame, at 425 minutes, indicates that the bipolar division has been maintained and therefore deemed successful. QLT (category 2): A QLT-0267-treated cell b attempts bipolar anaphase, resulting in lobes b1 and b2 at 71 minutes. These temporary cytoplasmic partitions subsequently collapse into each other giving rise to the cell labeled c at 119 minutes. Cell c becomes symmetrical by the last frame at 134 minutes. DMSO (Category 3): A control cell treated with drug vehicle (labeled d) elongates beginning bipolar cytokinesis (frame 2, 555 minutes) resulting in 2 lobes (d1 and d2) at 591 minutes (frame 3). These lobes subsequently collapse into one cell (labeled e) at 639 minutes. By 691 minutes (frame 5) the integrity of the cell membrane is lost and the cell dies leaving only debris (frame 6–7, 694–708 minutes). DMSO (Category 4a): A cell labeled f, treated with DMSO, initiates a multipolar division, giving rise to 5 lobes (f1-f5). Lobes f4 and f2 collapse together forming lobe g and lobes f5 and f1 collapse together to form lobe h. Lobes g and h (frame 4 and 5, 346–357 minutes) collapse to form lobe i that collapses with lobe f3 to form the cell labeled “j” (frame 7, 366 minutes). Ensuing death of cell j is clearly evident over an hour later (frame 9, 463 minutes). Only cellular debris was observed in the final frame. QLT (Category 4b) represent a multipolar division by a cell exposed to the ILK inhibitor that ultimately results in one viable cell. Four lobes (k1-k4) can be seen at 186 minutes (frame 4). Lobes k2 and k3 subsequently split into two additional lobes (k2a,b and k3a,b) at 200 minutes. Lobes k1 and k4 collapse to form lobe l, followed by the lobes l and k3b collapsing to form lobe m. At 282 minutes (frame 9), lobes m and k3a collapse to form n. The three remaining lobes collapse at 320 minutes resulting in a single cell that remains viable for over an hour. QLT (Category 4c) Represents an attempted multipolar division by a cell exposed to QLT-0267 that ultimately results in two viable cells. Cytoplasmic division of a cell labeled p results in 5 lobes (p1-p5, 72 minutes). Over the course of the next two hours, successive collapse involving lobes p1, 2, 4, and 5 results in two, new viable cells (one generated from p3 and the other generated from the remaining lobes). (B) A z-stack data set of 151 layers at a 0.16 µm step was taken of a Y79 cell attempting multipolar cytokinesis in the presence of 10 µM QLT-0267. Z positions of the z-stack are indicated. Irregular constrictions were observed in this mitotic cell that divided the cytoplasm into separate lobes. **(Bottom Panel)** Arrowheads indicate an area where the cell cortex is interrupted and arrows indicate missorted clusters of chromosomes. (C) An increase in the percentage of viable multinucleate cells was observed in the presence of QLT-0267. **(Left)** The total number of multinucleated cells is represented as a percentage of the total number of cells in QLT-0267 and DMSO treated groups following a 4–5 day exposure to drug or vehicle control. A significant increase in the percentage of multinucleated cells was observed in QLT-0267 treated cells as compared to control. Bars represent mean ± SEM, n = 6. * p<0.05 as determined by Student's t-test. **(Right)** In the same trials used to assess multinucleated cells, Y79 cells were assayed for viability using a Propidium Iodide (PI) exclusion assay. PI positive, multinucleated cells were quantitated. This was expressed a percentage of control of total multinucleated cells. Bars represent mean ± SEM, n = 6. * p<0.05 as determined by Student's t-test.

To examine the possibility that ILK inhibition gives rise to multinucleated cells as a result of failed cytokinesis, we monitored the behavior of dividing cells in time-lapse videos. Y79 cells were observed by DIC and the fate of all cells undergoing mitosis categorized into 4 groups (Fig. 6A). Failed multipolar cytokinesis was further divided into 3 subcategories (4a-c). Subcategories represent the number of cells that were produced by multipolar divisions and the number of multipolar divisions leading to death. Multipolar divisions not leading to cell death were categorized as “failed” if their polarity of division (number of poles in a multipolar mitosis) during anaphase was greater than the number of cells produced. It is noteworthy that multipolar cytokinesis leading to death did not occur in retinoblastoma cells treated with the ILK inhibitor while 16% of cells undergoing multipolar cell division in DMSO treated cultures died (see Category 4a). However, a much higher percentage of multinucleated cells in QLT-0267 treated cells, exited mitosis without completing cytokinesis as compared to controls (4b: 16% versus 4%, respectively). This occurred at the final stages of cytokinesis and is consistent with earlier reports demonstrating an increased frequency of multipolar divisions that resulted in fewer daughter than spindle poles in ILK inhibited cells[16]. Successful bipolar division was reduced in QLT-0267 treated Y79 cells as compared to DMSO controls (31% versus 54%, respectively) while failed bipolar cytokinesis was increased (51% versus 19%, respectively). Like multipolar cytokinesis, a decrease in cell death following bipolar mitosis was observed in QLT-0267 treated cultures. This finding differs from earlier reports in MDA-MB-231 cells (wild-type for the *Rb* gene product) where apoptosis following the arrested mitosis was increased by ILK inhibition [16]. Together this effect on failed bipolar and multipolar cytokinesis may explain the increase in multinucleated cells seen in QLT-0267 treated cultures over time. A propidium iodide (PI) exclusion assay was performed on QLT-0267 treated and untreated cultures. As with time-course analysis of retinoblastoma cell lines treated with the inhibitor, an increase in the total cell number of multinucleated cells was observed (Fig 6C, left bar graph). In these same trials, Y79 multinucleated cells were assayed for viability. Viability in drug and vehicle treated cells were not significantly different (Fig. 6C, right bar graph).

## Discussion

### ILK and Centrosome Clustering

Supernumerary centrosomes refer to cells carrying more than the normal compliment of centrosomes, a phenomenon commonly seen in cancer cells [26]. Tumour cells survive with extra chromosomes by developing mechanisms to cluster supernumerary centrosomes promoting chromosome segregation on bipolar spindles, thereby ensuring bipolar mitosis and cell survival [27], [28]. Alternatively, disruption of clustering leads to multipolar mitotic spindle formation and ensuing death. For this reason, disruption of clustering is a strategy for cancer therapies as it is thought to selectively target cancer cells bearing supernumerary centrosomes. During cell division, ILK has been shown to regulate microtubule dynamics and centrosome clustering. In both cell lines that express normal levels of the *Rb* gene product (i.e. HEK 293, HeLa, MDA-MB-231, BT 549, PC3) and those that do not (i.e. U87) ILK inhibition has resulted in either mitotic arrest or de-clustered centrosomes [15], [16], [21], [22]. In these studies, exposure times varied from 7 hours to 48 hours. Time-lapse microscopy using MDA-MB-231 (breast cancer cells wild-type for the *Rb* gene product) indicate that ILK inhibition prevented successful bipolar division, resulting in mitotic arrest and either subsequent exit from mitosis or cell death [16]. Although a modest increase in cytokinesis failure was reported in the presence of the ILK inhibitor, an increase in multinucleated cells was not observed. In contrast, data from our lab indicate that ILK inhibition markedly increased the percentage of multinucleated retinoblastoma cells, an effect that correlates with altered mitotic spindle organization and failed cytokinesis. In contrast to earlier reports, in Rb deficient retinoblastoma cell lines, ILK inhibition decreased cell death following the initiation of multipolar division, increasing the accumulation of multinucleated cells over time. Also, although earlier reports did observe successful ¾ way cytokinesis, we did not observe successful multipolar division in retinoblastoma cells. Rather, all viable multipolar divisions were categorized as failed because their polarity of division during anaphase was greater than the number of cells produced (the maximum number of cells produced following failed multipolar division was two).

One key difference exists between these cellular paradigms namely: retinoblastoma cells used in this study lack normal Rb protein levels while MDA-MB-231 cells are wild-type for the *Rb* gene product. Furthermore, QLT-0267 exposure times were considerably shorter in other studies that used cells lacking normal Rb protein expression. Shorter exposure times might have been insufficient to observe mitotic changes in a subpopulation of cells culminating in the gradual accumulation of a significant number of multinucleated cells. If multinucleation does indeed occur in cell lines lacking *Rb* gene expression, how can Rb-deficient cells override checkpoint arrest and undergo karyokinesis? Cytokinesis is a highly ordered process requiring an intricate interplay between cytoskeletal and cell signaling pathways. Additional cellular processes are also required including membrane trafficking. Cytokinesis failure can lead to the production of multinucleated cells and to centrosome amplification. ILK appears to alter centrosome clustering through microtubule regulating proteins TACC3 and ch-TOG and Aurora-A kinase (a kinase known for its vital role in spindle organization). Indeed we have observed supernumary centrosomes in retinoblastoma cells. Centrosome clustering is altered by ILK whether or not the *Rb* gene product is expressed as this was observed in breast and prostate cancer cells (expressing normal Rb protein levels) [16] and in retinoblastoma lines (devoid of Rb protein expression). However, the ultimate outcome of this de-clustering may depend upon the integrity of the Rb pathway. It would be interesting to determine whether or not long-term exposure to QLT-0267 in other Rb-deficient cell lines increases multinucleation and whether or not expressing the Rb gene product in Rb negative cells would ameliorate the effects of ILK on multinucleation. Of course the Rb protein, though expressed, may be functionally inactivated as this is common in human cancer lines. Functional Rb deficiency may arise from multiple mechanisms (not just through loss of Rb expression) including the altered expression or activity of Rb pathway regulators and DNA tumour viral oncoproteins that bind and inactivate Rb [29], [30]. Care in the design of these experiments would need to be implemented to ensure that a comparison was made between cells with and without a fully functional Rb pathway. Attention to this is especially significant given that integrity of the Rb pathway may be critical for directing therapy of certain forms of cancer [31].

Polyploid animal cells with supernumerary centrosomes can have various fates that include: 1) cell cycle arrest and apoptosis; 2) multipolar mitosis ending up in aneuploidy that may end up in cell death and 3) successful bipolar mitosis through the clustering of supernumary centrosomes to form bipolar spindles. The multipolar cells undergoing mitosis in the presence of ILK downregulation may be undergoing mitotic catastrophe or anaphase catastrophe two different mechanisms that result from decreased mitotic fidelity. Although poorly understood, mitotic catastrophe originates from aberrations in the mitotic apparatus that is accompanied by some degree of mitotic arrest [27]. Mitotic catastrophe is also referred to as mitotic slippage because cells slip out of mitosis without satisfying the requirements of the spindle assembly checkpoint [28]. Alternatively, anaphase catastrophe satisfies the spindle assembly checkpoint and only initiates chromosome separation after chromosomes are attached to the spindle. Indeed some have proposed that ILK inhibition promotes multipolar anaphases that trigger anaphase catastrophe [28]. Both mechanisms underly compromised mitotic fidelity and are thought to be oncosuppressive in that they ultimately result in cell death or cell senescence. Rb-deficient cells have dysregulated G1 checkpoint and cell cycle exit [10], [11]. Following ILK downregulation, if Rb-deficient cells are able to “cheat death” following chromosomal segregation to two or more “daughter” nuclei without cytokinesis then multinucleated cells would be expected to increase. Centrosome impairment and cytokinesis defects are proposed mechanisms of other anticancer drugs [32]. Multinucleated cells have only been observed in a retinoblastoma and in an irradiated model of retinoblastoma grown in nude mice. Both were thought to be undergoing tumour regression [33]. Therefore, increased multinucleation observed when ILK is downregulated may be a unique anticancer mechanism ultimately leading to the gradual elimination of multinucleated cancer cells and to tumour regression.

## Materials and Methods

### Cell Culture and Drug Exposure

Human retinoblastoma cell lines Y79, Rb143 and Weri-Rb 27 were grown in suspension (unless stated otherwise) and cultured using DMEM+Glutamax, with 10% fetal bovine serum and gentamicin (50 µg/ml). All cell lines were originally derived from primary tumor explants recovered from the enucleated eye of a patient [34]. Cells were incubated at 37°C and 5% CO_2_. High density cultures were split 1∶2 every one to two days while low density cultures were split 1∶10 once a week. Cells were treated with a highly specific small molecule inhibitor for ILK (QLT-0267, Valocor Therapeutics, Inc., Vancouver, BC, Canada). Stock QLT-0267 was suspended in DMSO at a concentration of 25 mM and stored at -20°C. Cells were exposed to QLT-0267 (or vehicle control) in complete media containing 10% fetal bovine serum.

### Transfections (siRNA and FLAG Constructs) and Transductions (shRNA)

ILK-specific and control siRNA constructs (5′-GACGCTCAGCAGACATGTGGA-3′) were purchased from Qiagen (Mississauga, ON, Canada) and used with Qiagen's HiPerFect reagent to transfect Y79 cells according to the manufacturer's protocol and previously published manuscripts [14], [16]. Cells were transfected at 30,000 cells/well in 24-well plates (600 µL volume); siRNA was used at 17nM in Opti-MEM media (Life Technologies Inc., Burlington, ON, Canada). Cells were re-transfected two days after the initial transfection, and analyzed four days after the initial transfection (unless otherwise stated). Knockdown efficacy was confirmed by immunocytochemistry and computerized fluorescence intensity analysis. Y79 cells were transfected with FLAG-tag or FLAG-ILK (wild-type) plasmids [35] in Opti-MEM with Lipofectamine 2000 transfection reagent (Life Technologies) following the manufacturer's instructions. Positive clones were selected in the presence of 300 µg/ml Geneticin for at least 10 days. Expression of FLAG-ILK was confirmed by Western blotting. Y79 cells were transduced with ILK shRNA and control shRNA Lentiviral Particles previously used in other human cell lines [36] according to the manufacturer's protocol (SantaCruz, Biotechnology, Dallas, TX, USA). Stable cells expressing the shRNA were isolated in 1 µg/ml puromycin containing media.

### Western Blotting

Western blotting was used to confirm ILK knockdown in Y79 cells treated with siRNA. Cells were lysed in RIPA buffer containing protease inhibitors (Complete-protease inhibitor tablets; Roche Applied Science, Laval, QC, Canada), 1 mM PMSF, 2mM NaF and 1 mM Na_3_VO_4_.and Western blots were run as previously described [37]. Membranes were probed first with a monoclonal anti-ILK antibody (1∶1000; BD Biosciences Mississauga, ON, Canada), stripped using Restore™ buffer (Thermo Fisher Scientific, IL, USA) and subsequently reprobed with a rabbit anti-Gapdh antibody (1∶200; Santa Cruz, TX, USA) as a control for total protein expression. To confirm the expression of FLAG-ILK in transfected lines, membranes were probed first with a monoclonal antibody to FLAG (1∶200; Sigma-Aldrich anti-FLAG M2), stripped and subsequently reprobed with an anti-Gapdh (as stated above). Proteins were detected using Biorad Clarity Western ECL substrate and visualized by Biorad ChemiDoc XRS+ with Image Lab Software (Mississauga, ON, Canada).

### Quantitative PCR (Q-PCR)

RNA, obtained using GeneJet RNA purification Kit # K0731 (Fermentas, Thermo Scientific, Ottawa, ON, Canada) from Y79 cells, was reverse transcribed using Revert Aid H minus First cDNA synthesis Kit # K1632 using random hexamers according to manufacturers instructions (Fermentas, Thermo Fisher Scientific). The resulting first-strand cDNA was used as template for the real-time Q-PCR. The Applied Biosystems 5700 Sequence Detection System (Perkin-Elmer Applied Biosystems, Foster City, CA, USA) was used for real-time monitoring of PCR amplification of cDNA using the SYBRO Green Universal PCR Master Mix protocol. Amplification of the following cDNAs was performed using the following primers: ILK (F): CGGCTCAGGATTTTCTCGC and (R): GGTCCACGACGAAATTGGTG. Relative quantification of gene expression was performed using β-actin as a control. β-Actin cDNA was amplified separately on a duplicate set of samples using standard primers from Invitrogen, Life Technologies.

### Immunocytochemistry

Y79 and Rb143 cells grown in suspension were adhered to poly-D-lysine coated 12-well slides, after which they were fixed for 10 minutes at room temperature in 4% paraformaldehyde. Cells were then permeabilized using 0.2% Triton/TBS and blocked with 5% NGS in 0.1% BSA/TBS-Tween. The cells were then incubated overnight at 4°C with antibodies against ILK (Abcam Burlington, ON, Canada, rabbit, 1∶200), α-tubulin (Sigma-Aldrich, mouse, 1∶1000), pericentrin (Abcam, rabbit 1∶1000; Abcam, mouse, 1∶100), γ-tubulin (Abcam, rabbit, 1∶1000), and β-tubulin III (Chemicon International, Billerica, MA, mouse, 1∶500), diluted in 0.1% BSA/TBS-Tween containing 1% NGS. Cells were then washed and incubated with secondaries. Alexa fluor secondary antibodies anti-mouse 488 (Invitrogen, Burlington, ON, Canada, 1∶200), anti-mouse 568 (Invitrogen, 1∶200), anti-rabbit 488 (Invitrogen, 1∶200) and anti-mouse 568 (Invitrogen, 1∶200) were used to detect antigen-antibody binding. Cells were then stained for Hoechst 33342, washed and mounted using Vectashield mounting media (Vector Laboratories, Burlington, ON, Canada). F-actin was detected using Alexa Fluor 568 Phalloidin (Invitrogen) or Alexa Fluor 488 Phalloidin (Invitrogen) and was used during immunocytochemistry in conjunction with fluorescent secondary antibodies.

### Viability Assay

To assay for viability, cells were incubated in DMEM containing 2% FBS and 2 µg/mL propidium iodide for 30 minutes at 37°C, washed with PBS, and fixed with 4% paraformaldehyde. The number of propidium iodide-positive cells was calculated as a percentage of total cell number (Hoechst-stained cells).

### Flow Cytommetry

Cells were rinsed with PBS and fixed with ice cold 70% ethanol. Cells were subsequently stained with a propidium iodide staining buffer (0.1 mg/ml RNase A, 0.05% Triton-X-100 and 50 µg/ml in PBS) for 1 hour. Samples were analyzed using a BD FACS Canto II flow cytometer and BD FACSDiva or Flo Jo software.

### Microscopy and Live-Imaging

Cells were viewed under an Olympus IX81 inverted microscope equipped with the Olympus DSU (Disk Scanning Unit) spinning disk confocal. Images taken using the Olympus IX81 inverted microscope, were analyzed using ImageJ and Metamorph Premier software. Nuclear size was analyzed using wide-field imaging while nuclear number was determined by analyzing z-stacks of fluorescent images. For live-imaging, cells were preexposed to 10 µM QLT-0267 or vehicle control for 24 hours. Cells were then plated onto poly-D-lysine coated glass-bottomed tissue culture dishes, in plating media containing the drug or vehicle. The stage temperature was controlled by a PrecisionControl Weather Station and the humidified chamber housing the cells was kept at 37°C with 5% CO_2_. After the cells had adhered, pictures were taken at low light every 1.5 minutes over 30 adjacent fields of view for a 12 hour period. For the representative individual frames, contrast and brightness of the images was adjusted in ImageJ.

### Immunofluorescence Quantification

Metamorph was used for quantification of fluorescence intensity of ILK at the centrosome. A threshold was determined, allowing only the brightest of pixels (5%) to be visible in a given field of view in the control siRNA. The average pixel intensity of ILK at the centrosomes above this predetermined threshold was quantitated for a minimum of 500 cells/trial, n = 3.
